# Play Turned Painful: A Teenager’s Tibial Pilon Fracture from A Simple Jump

**DOI:** 10.5811/cpcem.35491

**Published:** 2025-01-26

**Authors:** Victoria M Koniuk, Brody M Fogleman, Lindsay Tjiattas-Saleski

**Affiliations:** Edward Via College of Osteopathic Medicine – Carolinas, Department of Emergency Medicine, Spartanburg, South Carolina

**Keywords:** tibial pilon fracture, tibial plafond fracture, low-energy trauma, open reduction internal fixation

## Abstract

**Case Presentation:**

An 18-year-old male presented with severe left ankle pain and inability to bear weight after jumping from a three-foot platform. Physical examination revealed decreased range of motion of the left ankle without visible deformity or neurovascular deficits. Imaging studies showed a vertical fracture of the distal tibia—a pilon fracture without fibular involvement.

**Discussion:**

Pilon fractures involve the distal tibial articular surface and are rare. They typically result from high-energy trauma and often involve the fibula. This case illustrates a low-energy mechanism resulting in a pilon fracture without fibular involvement in a young patient without typical risk factors. It highlights the importance of considering pilon fractures in low-energy ankle injuries and the need for appropriate management even in less-complex cases.

## CASE PRESENTATION

**Image 1:**
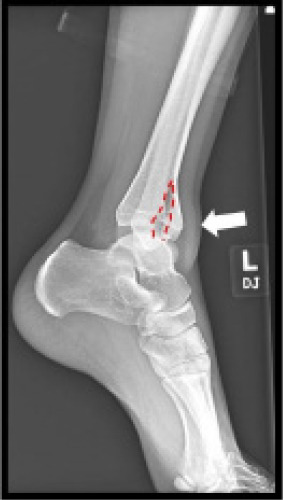
Lateral view plain film reveals a vertical fracture of the distal tibia (red dashed line) and soft-tissue swelling (white arrow).

**Image 2:**
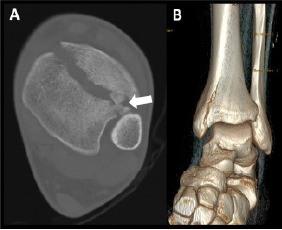
Computed tomography without contrast in the transverse plane shows a distal fracture line with bone fragments interposed, as indicated by the white arrow (A), and a three-dimensional rendering (B) reveals a fracture of the distal tibia without fibular involvement.

An 18-year-old male presented to the emergency department with severe pain and inability to bear weight on his left leg following a jump from a three-foot-high platform while playing an airsoft game. The patient remembered landing equally on both feet at the time of impact. Physical examination revealed decreased range of motion of the left ankle, no visible deformity, and intact neurovasculature. An ankle plain-film radiograph and follow-up computed tomography were obtained as seen in Image 1 and Image 2, respectively.

## DISCUSSION

Pilon fractures, also known as tibial plafond fractures, are a subtype of lower extremity fractures that involve the distal articular surface of the tibia, accounting for less than 1% of all lower extremity fractures^1^ and 7–10% of all tibial fractures.^2^ Approximately 90% of pilon fractures are associated with concomitant fibula fractures.^3^ Mechanisms of injury are most often high-energy, axial loading^1^ injuries causing the talus to advance distally resulting in impaction and comminution of the tibial metaphysis.^4^ Although low-energy mechanisms of injury are less common, studies have postulated that risk factors include increased age and a history of osteoporosis, neither of which were applicable to this patient.^5^

Although current management strategies are based upon research focusing on highly comminuted fibula-involving pilon fractures, there is less evidence on appropriate management of low-energy, fibula-sparing cases. Current literature shows that splinting or external fixation along with orthopedic consultation is the necessary initial management.^5^ This case emphasizes the importance of including pilon fractures in a differential diagnosis and investigating appropriate management for low-energy, less-complex fracture subtypes, even when the affected patient’s demographics do not necessarily match the associated risk factors for this condition.

**CPC-EM Capsule**

What do we already know about this clinical entity?

*Pilon fractures are rare tibial fractures involving the distal articular surface, typically from high-energy trauma, often with fibula fractures*.

What is the major impact of the image(s)?

*The lateral plain film and computed tomography reveal a vertical tibial fracture without fibular involvement, offering a visual representation of a low-energy pilon fracture*.

How might this improve emergency medicine practice?

*This case emphasizes the need to consider pilon fractures in low-energy trauma, the importance of imaging for appropriate management, and highlights unusual demographics*.
